# Zn_2_GeO_4_@CeO_2_ Core@Shell Nanorods for Efficient Photocatalytic CO_2_ Reduction

**DOI:** 10.3390/molecules30102205

**Published:** 2025-05-18

**Authors:** Jin Sun, Yunxia Bai, Xilan Feng, Dapeng Liu, Yu Zhang

**Affiliations:** 1 Hangzhou International Innovation Institute, Beihang University, Hangzhou 311115, China; 20271015@buaa.edu.cn (J.S.); baiyunxia@buaa.edu.cn (Y.B.); jade@buaa.edu.cn (Y.Z.); 2 School of Chemistry, Beihang University, Beijing 100191, China; 3 School of Chemistry and Chemical Engineering, Shaoxing University, Shaoxing 312000, China; 4 Zhejiang Key Laboratory of Functional Ionic Membrane Materials and Technology for Hydrogen Production, Shaoxing 312000, China

**Keywords:** CO, CO_2_ reduction, CeO_2_, photocatalysis, Zn_2_GeO_4_

## Abstract

The enduring problem of CO_2_ emissions and their consequent influence on the earth’s atmosphere has captured the attention of researchers. Photocatalytic CO_2_ reduction holds great significance; however, it is constrained by the effect of carrier recombination. Simultaneously, the structural modification of heterojunction catalysts has emerged as a promising approach to boost the photocatalytic performance. Herein, Zn_2_GeO_4_@CeO_2_ core@shell nanorods were prepared by a simple self-assembly method for photocatalytic CO_2_ reduction. The thickness of the CeO_2_ shell can be regulated rapidly and conveniently. The photocatalytic results indicate that the structure regulation could affect the photocatalytic performance by controlling the amount of active sites and the shielding effect. X-ray photoelectron spectroscopy (XPS) and Mott–Schottky analyses reveal that Zn_2_GeO_4_ and CeO_2_ formed Type-I heterojunctions, which prolonged the lifetime of the photogenerated carriers. The CO_2_ adsorption and activation capacities of CeO_2_ also exert a beneficial influence on the progress of CO_2_ photoreduction, thus enabling efficient photocatalytic CO_2_ reduction. Moreover, the in situ FT-IR spectra show that Zn_2_GeO_4_@CeO_2_ suppresses the formation of byproduct intermediates and shows higher CO selectivity. The best sample of Zn_2_GeO_4_@0.07CeO_2_ can exhibit a CO yield of as high as 1190.9 μmol g^−1^ h^−1^.

## 1. Introduction

In recent decades, the combustion of fossil fuels has led to a substantial increase in the atmospheric concentration of CO_2_. As a greenhouse gas, an excessive CO_2_ concentration can trigger a series of environmental problems [1,2,3]. Considerable efforts have been devoted to reducing atmospheric CO_2_ levels such as photocatalysis, electrocatalysis and thermocatalysis. Among these, photocatalytic CO_2_ reduction into high-value-added products is regarded as an effective approach to mitigate energy problems and reduce the greenhouse effect [4,5,6]. A variety of semiconductors, including TiO_2_, CdS, and Zn_2_GeO_4_, have been employed as photocatalysts [7,8,9].

For photocatalytic CO_2_ reduction catalysts, the primary limitation lies in the intense photogenerated carrier recombination, which significantly impacts the lifetime of carriers [10,11,12]. In an attempt to surmount this constraint on carrier lifetime, heterojunction photocatalysts have been extensively investigated [13]. The photogenerated carriers in the heterojunctions can transfer between semiconductors, thus generating highly active charge carriers, which is advantageous for reducing the probability of recombination [14,15,16]. Currently, a wide variety of heterojunction photocatalysts with diverse morphologies and structures have been investigated, and their structure and morphology have emerged as a prevalent research focus [7,14,17,18]. It is generally accepted that the carrier transfer process predominantly takes place at the heterostructure interface [19]. In this case, heterojunction catalysts with a special core@shell structure, which possess a large interfacial area, have been widely studied [16]. Structure regulation of the core@shell heterojunction catalysts enables the components to leverage strengths to offset weaknesses, thereby achieving the optimal effect [17]. Moreover, controllable tuning is of great significance in catalyst design.

Zinc germanate (Zn_2_GeO_4_), owing to its good CO_2_ reduction capability, is widely utilized in the field of photocatalysis [20,21]. However, the wide band gap, low charge separation efficiency, and insufficient active sites of Zn_2_GeO_4_ impose limitations on its photocatalytic efficiency [22]. To remove these obstacles, various methods have been employed by researchers such as adjusting the oxygen vacancies concentration [9]. Cerium dioxide (CeO_2_) is an n-type semiconductor, and there are a substantial number of oxygen vacancies on its surface, which can act as reaction sites for the activation of CO_2_ [23,24,25]. Inspired by the above analysis, the construction of Zn_2_GeO_4_@CeO_2_ might prove to be an effective way to achieve an ideal photocatalytic effect.

In this work, Zn_2_GeO_4_@CeO_2_ was fabricated by a self-assembly method. The amount of the coated CeO_2_ shell was carefully controlled to adjust the amount of active sites. The as-obtained Zn_2_GeO_4_@CeO_2_ catalysts were then comprehensively characterized to identify the optimal amount of the CeO_2_ shell that exhibits the best performance on photocatalytic CO_2_ reduction. Mott–Schottky analysis and UV–visible spectroscopy (UV-vis) characterizations were conducted to reveal the reaction mechanism. The results suggest that the formation of Zn_2_GeO_4_@CeO_2_ nanorods could extend the carrier lifetime and facilitate the light absorption process, with their catalytic performance being significantly influenced by the coating thickness of the CeO_2_ layer. It is believed that this work can provide a novel strategy for the design of heterojunction catalysts for photocatalytic CO_2_ reduction.

## 2. Results and Discussion

**Figure 1:**
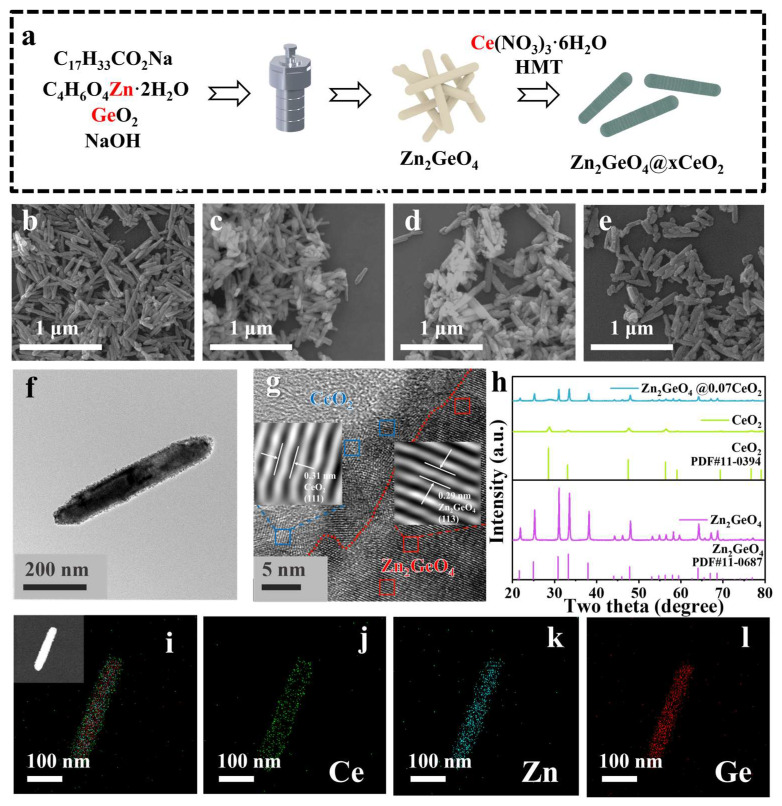
(**a**) Schematic synthesis of Zn_2_GeO_4_@xCeO_2_ samples; (**b**–**e**) SEM images of the as-obtained samples: (**b**) Zn_2_GeO_4_, (**c**) Zn_2_GeO_4_@0.05CeO_2_, (**d**) Zn_2_GeO_4_@0.07CeO_2_, and (**e**) Zn_2_GeO_4_@0.1CeO_2_; (**f**,**g**) HRTEM images of Zn_2_GeO_4_@0.07CeO_2_; (**h**) XRD patterns of Zn_2_GeO_4_, CeO_2_, and Zn_2_GeO_4_@0.07CeO_2_; (**i**–**l**) EDS images of Zn_2_GeO_4_@0.07CeO_2_.

The schematic synthesis process of a series of Zn_2_GeO_4_@CeO_2_ core@shell nanorods is presented in Figure 1a. Initially, the Zn_2_GeO_4_ precursor was synthesized via hydrothermal treatment, followed by CeO_2_ coating to form the core@shell architecture. A series of Zn_2_GeO_4_@CeO_2_ samples have been prepared, which are named as Zn_2_GeO_4_@xCeO_2_, where x represents the amount of Ce added per 50 mg of Zn_2_GeO_4_ (Figure 1b–e). Scanning electron microscopy (SEM) was applied to characterize the morphology of the series samples. As shown in Figure 1b and Figure S1, the as-obtained Zn_2_GeO_4_ shows a rod-shaped morphology. After coating, the surface of these nanorods became rough, implying the formation of the core@shell structure [26,27]. The high-resolution transmission electron microscopy (HRTEM) images of Zn_2_GeO_4_@xCeO_2_ in Figure 1f show that a layer of CeO_2_ nanoparticles could be observed clearly on the surface of Zn_2_GeO_4_. To further investigate the structure of the samples, Zn_2_GeO_4_@0.07CeO_2_ was chosen as the representative sample. As shown in Figure 1g, it can be observed that the lattice spacings of the core and shell of 0.29 and 0.31 nm correspond well to the characteristic (113) plane of Zn_2_GeO_4_ and the (111) plane of CeO_2_, respectively [20,23,28]. Figure 1h and Figure S4 present the X-ray diffraction (XRD) patterns of the samples, where all diffraction peaks of the as-obtained Zn_2_GeO_4_ and CeO_2_ could be well indexed to the standard patterns of Zn_2_GeO_4_ (JCPDS #11-0687) and CeO_2_ (JCPDS #11-0394). The intensities of the signals corresponding to Zn_2_GeO_4_ decrease when compared to those of Zn_2_GeO_4_, which should be attributed to the encapsulation of the CeO_2_ shell. However, the intensities of the characteristic peaks of CeO_2_ gradually increase as the Ce dosage increases [8]. Therefore, as shown in Figure 1i–l and Figure S5, energy dispersive X-ray spectroscopy (EDS) mapping analysis shows that the elements Zn and Ge are present in the core position; however, Ce is only distributed on the nanorod surface. Furthermore, the thickness of the CeO_2_ shell increases with the increase in CeO_2_ (Figures S5 and S6).

**Figure 2:**
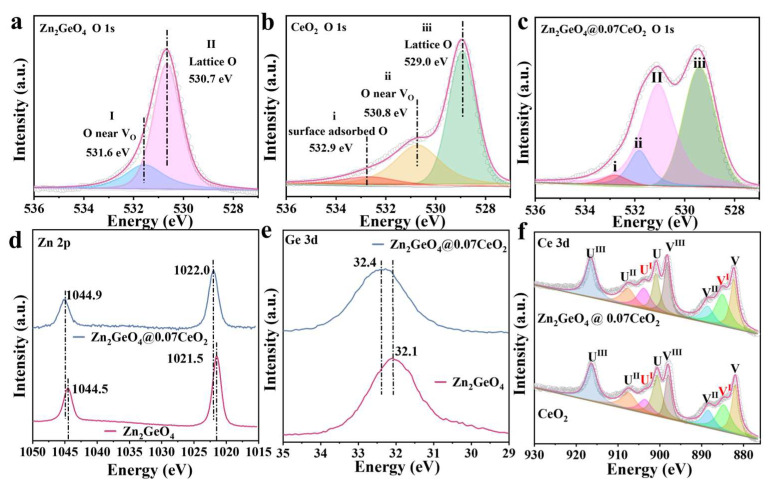
O 1s XPS spectra of (**a**) Zn_2_GeO_4_, (**b**) CeO_2_, and (**c**) Zn_2_GeO_4_@0.07CeO_2_; (**d**) Zn 2p XPS spectra of Zn_2_GeO_4_@0.07CeO_2_ and Zn_2_GeO_4_; (**e**) Ge 3d XPS spectra of Zn_2_GeO_4_@0.07CeO_2_ and Zn_2_GeO_4_; (**f**) Ce 3d XPS spectra of Zn_2_GeO_4_@0.07CeO_2_ and CeO_2_.

X-ray photoelectron spectroscopy (XPS) was employed to identify the surface chemical environments of the as-obtained samples. As shown in Figure 2a, the peaks centered at 530.7 and 531.6 eV can be assigned to the lattice oxygen and oxygen atoms near oxygen vacancy in Zn_2_GeO_4_ [29,30,31]. For CeO_2_, the O 1s XPS peak at 532.9 eV can be ascribed to the adsorbed (CO_2_ or H_2_O) oxygen on the surface of CeO_2_, indicating the outstanding CO_2_-storing ability (Figure 2b) [26,32,33]. The significant peak at 530.8 eV can be attributed to the oxygen atoms near oxygen vacancy defects. It is further substantiated by the Electron Paramagnetic Resonance (EPR) tests that the signal of oxygen vacancy at g = 2.002 can be observed (Figure S7). The abundant O vacancies in CeO_2_ could act as active sites for photocatalytic CO_2_ reduction [24]. All the peaks displayed in the O 1s signals mentioned above have been included in Zn_2_GeO_4_@0.07CeO_2_ (Figure 2c).

Moreover, the electron binding energy of the element originates from the Coulomb attraction between the outer electrons of the atom and the atomic nucleus, in which a chemical shift can directly reflect the changes in electron density [17,34,35]. For the XPS spectra of Zn 2p and Ge 3d, the electron transfer process after the formation of the heterojunction was characterized by comparing Zn_2_GeO_4_ with Zn_2_GeO_4_@0.07CeO_2_ (Figure 2d,e). As shown in Figure 2d, the peaks located at 1021.5 and 1044.5 eV can be fitted into Zn 2p_3/2_ and Zn 2p_1/2_, respectively. In Figure 2e, the peaks centered at 32.1 eV can be assigned to Ge 3d [20,29]. Compared to Zn_2_GeO_4_, Zn 2p_3/2_ and Zn 2p_1/2_ in Zn_2_GeO_4_@0.07CeO_2_ shift to 1022.0 and 1044.9 eV, and Ge 3d shifts to 32.4 eV, which could be attributed to the electron transfer from Zn_2_GeO_4_ to CeO_2_ [23]. In Figure 2f, the Ce 3d XPS spectra can be deconvoluted into eight peaks denoted as V and U, ascribed to the spin−orbit coupling of the Ce 3d_5/2_ and Ce 3d_3/2_ states, respectively, which also correspond to Ce^3+^ and Ce^4+^ [26,36,37]. The presence of Ce^3+^/Ce^4+^ redox couples can create oxygen vacancies, which can serve as active sites and enhance the photocatalytic activity [25]. As a result, the Ce^3+^ content in Zn_2_GeO_4_@0.07CeO_2_ is 35.7%, which is significantly higher than the 25.8% in pure CeO_2_ (Table S1, Supporting Information). This can be mainly attributed to the electron transfer from Zn_2_GeO_4_ to CeO_2_ [37,38].

**Figure 3:**
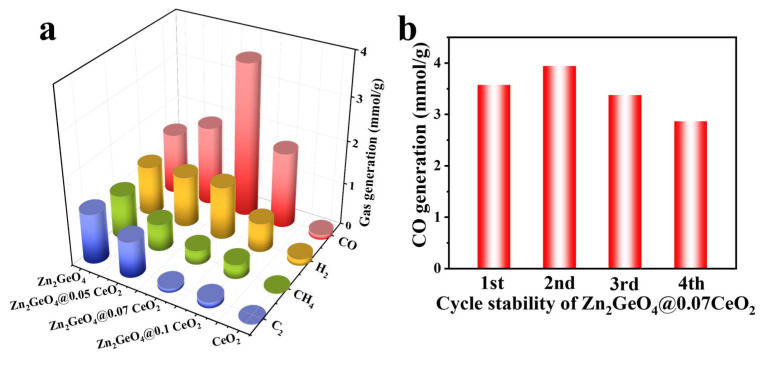
(**a**) Yields of products for different samples; (**b**) CO yields of Zn_2_GeO_4_@0.07CeO_2_ after four cycles.

Photocatalytic CO_2_ reduction was then conducted under full-spectrum irradiation and room temperature to evaluate the catalytic activities of the samples. As shown in Figure 3a, CO, H_2_, CH_4_, and C_2_ are the major gas-phase products. The CO yield of Zn_2_GeO_4_ is 380.1 μmol g^−1^ h^−1^, but the selectivity is only 10.0%. Among the samples, Zn_2_GeO_4_@0.07CeO_2_ has the highest CO yield of 1190.9 μmol g^−1^ h^−1^, which is 3.1 times that of Zn_2_GeO_4_, while its CO selectivity reaches 51.5% (Table S2). For Zn_2_GeO_4_, Zn_2_GeO_4_@0.05CeO_2_, and Zn_2_GeO_4_@0.07CeO_2_, the CO generation rates increase with the increase in CeO_2_, owing to the fact that the active sites on CeO_2_ near the interface are crucial for the reaction. The further increase in the thickness of CeO_2_ would result in a decline in catalytic performance, which can be attributed to the shielding effect, where the excessively thick CeO_2_ restricts the access of CO_2_ to the heterojunction interface, thereby impeding its reduction process [39]. ^1^H nuclear magnetic resonance (NMR) analysis indicates that negligible amounts of liquid-phase products were identified was detected in the system (Figure S8). As shown in Figure S9, the CO yield of Zn_2_GeO_4_@0.07CeO_2_ drops to 10.9 μmol g^−1^ h^−1^ in the absence of a sacrificial agent, comparable to that reported in the literature [29]. It is found that only a negligible amount of CO products are detected when no catalysts or external light source is present, further validating the origin of the product. Zn_2_GeO_4_@0.07CeO_2_ exhibits a similar CO yield over four cycles (Figure 3b). Moreover, there is no significant morphological change in Zn_2_GeO_4_@0.07CeO_2_ after reaction (Figure S10). A comparison of the XRD and XPS spectra of Zn_2_GeO_4_@0.07CeO_2_ before and after reaction indicates that there is no significant change, further demonstrating the structural stability of Zn_2_GeO_4_ (Figures S11 and S12).

**Figure 4:**
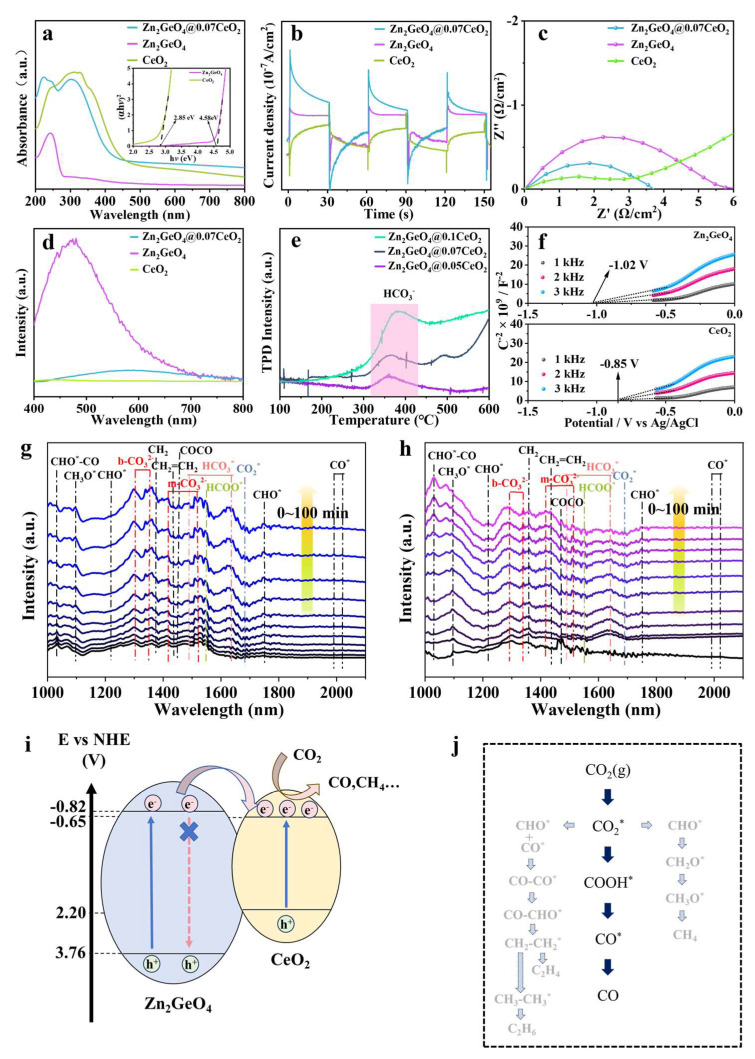
(**a**) UV-vis and Tauc spectra of Zn_2_GeO_4_ and CeO_2_; (**b**) transient photocurrent of Zn_2_GeO_4_@0.07CeO_2_; (**c**) EIS curves of Zn_2_GeO_4_@0.07CeO_2_; (**d**) PL spectra of Zn_2_GeO_4_@0.07CeO_2_; (**e**) CO_2_-TPD plots of Zn_2_GeO_4_@xCeO_2_; (**f**) Mott–Schottky curves of Zn_2_GeO_4_ and CeO_2_; in situ FT-IR spectra for CO_2_ photoreduction on (**g**) Zn_2_GeO_4_@0.07CeO_2_ and (**h**) Zn_2_GeO_4_; (**i**) band structures of Zn_2_GeO_4_ and CeO_2_ (pH = 7, vs. NHE); (**j**) reaction pathway of photocatalytic CO_2_ reduction.

The UV-vis spectra depict the light absorption capacity of the samples. Among them, Zn_2_GeO_4_@0.07CeO_2_ exhibits two absorption edges, which can be respectively attributed to Zn_2_GeO_4_ and CeO_2_ (Figure 4a). The formation of heterojunctions expands the absorption spectrum, thereby enhancing the light absorption performance of Zn_2_GeO_4_. In addition, the results demonstrate that the absorption edge of Zn_2_GeO_4_@0.07CeO_2_ undergoes a slight red shift as the amount of CeO_2_ increases, which can be attributed to the bandgap narrowing caused by the gradual enhancement of the absorption contribution of CeO_2_ (Figure S13). As shown in Figure 4b, Zn_2_GeO_4_@0.07CeO_2_ demonstrates a higher photocurrent density compared to Zn_2_GeO_4_ and CeO_2_, further confirming the highly efficient transfer of photogenerated charge and separation of carriers occurring on the Zn_2_GeO_4_@0.07CeO_2_ nanorods. Such efficiency can lead to more effective photogenerated electrons to enhance the ability of photocatalytic CO_2_ reduction [40]. Figure 4c demonstrates that Zn_2_GeO_4_@0.07CeO_2_ has a smaller electrochemical impedance spectroscopy (EIS) radius than that of Zn_2_GeO_4_. This also implies the effective separation and transfer of charge carriers in Zn_2_GeO_4_@0.07CeO_2_. The above results prove that the formation of Zn_2_GeO_4_@0.07CeO_2_ enhances the photocatalytic capability, which is also consistent with the results of the XPS analysis [35,41,42]. The photoluminescence (PL) intensity reveals the defect emission of Zn_2_GeO_4_, indicating a strong effect of carrier recombination [43]. In comparison, the decrease in the PL of Zn_2_GeO_4_@0.07CeO_2_ demonstrates the extension of the carrier lifetime [15]. Moreover, the CO_2_ temperature-programmed desorption (TPD) reflects that the adsorbed HCO_3_^−^ peak area increases with the increase in CeO_2_ (Figure 4e). This suggests that CeO_2_ provides abundant active sites for CO_2_ adsorption and activation [24,44,45].

The band structure of Zn_2_GeO_4_ and CeO_2_ was conducted by Mott–Schottky spectrum measurements. The plots of Zn_2_GeO_4_ and CeO_2_ exhibit positive slopes, suggesting that they are n-type semiconductors (Figure 4f) [7,17,44]. The flat-band potentials (E_fb_) of Zn_2_GeO_4_ and CeO_2_ are −0.82 and −0.65 V (vs. normal hydrogen electrode (NHE)), respectively [9,23,46,47]. The E_fb_ could roughly be equal to the conduction band (CB) potential of an n-type semiconductor [7,17,18]. Moreover, the band gaps (Eg) of Zn_2_GeO_4_ and CeO_2_ are determined by the UV-vis spectra and Tauc plots (Figure 4a), which are 4.58 eV and 2.85 eV, respectively [47,48]. Therefore, the valence band maximum (VBM) potentials of Zn_2_GeO_4_ and CeO_2_ are 2.20 and 3.76 V (vs. NHE), respectively. Due to the standard reduction potential of CO_2_/CO being −0.52 V (vs. NHE at pH = 7), the photocatalytic CO_2_ reduction can thermodynamically proceed under the photocatalysis of Zn_2_GeO_4_ and CeO_2_, and this is consistent with the experimental results [1,4]. The band structures in Figure 4i show that Zn_2_GeO_4_ and CeO_2_ should be ascribed to Type-I heterojunction. Under light excitation, the electrons of Zn_2_GeO_4_ and CeO_2_ will be excited from the valence band to the conduction band, respectively [42]. Due to the different conduction band energies, the photogenerated electrons with higher energy on the conduction band of Zn_2_GeO_4_ will migrate to the conduction band of CeO_2_, thus extending the carrier lifetime. The carriers will accumulate at the heterojunction interface of CeO_2_. Furthermore, the migration rate of holes is generally about two orders of magnitude slower than that of electrons. This effectively reduces the probability of carrier recombination [11,49]. The formation of heterojunction endows Zn2GeO4@0.07CeO2 with superior catalytic performance (Tables S3 and S4) [50,51,52,53,54,55,56,57,58,59,60].

To further clarify the reaction mechanism, in situ FT-IR measurements were performed on Zn_2_GeO_4_@0.07CeO_2_ and Zn_2_GeO_4_ during the CO_2_ photoreduction process. As shown in Figure 4g, the peak attributed to COOH^*^ was detected at 1550 cm^−1^ [61]. The COOH^*^ species are considered to be a key intermediate during CO_2_ reduction to CO. The bands located at 1478 cm^−1^ and 1637 cm^−1^ can be assigned to HCO_3_^*^ species [9]. A negative peak corresponding to CO_2_^*^ can be observed at 1683 cm^−1^, which can be attributed to the CO_2_ consumption by Zn_2_GeO_4_@0.07CeO_2_, demonstrating the high activity and fast reaction rates [62]. Furthermore, the distinct peaks observed at 1302 cm^−1^ and 1350 cm^−1^ can be assigned to the bidentate carbonate species (b-CO_3_^2−^), which indicates the abundant oxygen vacancies on the surface of Zn_2_GeO_4_@0.07CeO_2_ [62,63]. There will be a large amount of CO_2_ adsorbed near the oxygen vacancy of CeO_2_, and this adsorption is further enhanced due to the electron accumulation near the interface [24,62]. In contrast, Zn_2_GeO_4_ demonstrates more pronounced characteristic peaks indicative of intermediate species diversity, which correlates well with the poor selectivity. Moreover, Zn_2_GeO_4_ exhibits a certain capability to catalyze water splitting, thereby providing an abundant source of hydrogen species for the hydrogenation of adsorbed CO_2_ molecules, leading to the formation of CH_4_ [9,47]. Furthermore, due to the presence of asymmetric Zn-O-Ge sites, the CO* intermediates adsorbed on the Zn and Ge atoms exhibit different charge distributions, which facilitates C-C coupling [9]. As a result, Zn_2_GeO_4_ is more inclined to produce C_2_ products compared to CeO_2_. After coating with CeO_2_, the surface CeO_2_ serves as the adsorption and activation site for CO_2_ reduction, with CeO_2_ favoring the production of CO, thus altering the reaction pathway selectivity and reducing byproduct intermediates. Based on the in situ FT-IR spectra and the above analysis, the specific reaction pathway of Zn_2_GeO_4_@0.07CeO_2_ and Zn_2_GeO_4_ is depicted in Figure 4j [61,62,63,64,65].

## 3. Materials and Methods

### 3.1. Synthesis of Zn_2_GeO_4_

A total of 1100 mg of Zn(CH_3_COO)_2_·2H_2_O was added to 25 mL of deionized water and stirred magnetically for 10 min, and then 450 mg of GeO_2_ was added to the solution and stirred for 20 min. Next, 300 mg of sodium oleate (NaOA) was added slowly and stirred for another 20 min. Then, 5 mL of 3 mol L^−1^ NaOH aqueous solution was added dropwise to maintain the pH value at 7.5. After stirring for 20 min, the mixture was transferred to a 50 mL hydrothermal high-pressure reactor, sealed and heated at 140 °C for 24 h, and cooled naturally to room temperature. Then, the mixture was centrifuged and washed twice with 5 mol L^−1^ NaOH solution and dispersed into ethanol and water for washing. The sample was centrifuged five times and dried in a vacuum. Finally, the product was calcined in a N_2_ atmosphere at 400 °C for 3 h.

### 3.2. Synthesis of CeO_2_

A total of 1 mmol of Ce(NO_3_)_3_·6H_2_O was dissolved in a mixed solution of 20 mL of water and 20 mL of ethanol. Next, 25 mL of 0.02 g L^−1^ hexamethylenetetramine (HMT) solution was added. The mixture was heated to 70 °C, held for 2 h, and then cooled to room temperature. The product was purified by centrifugation, washed three times with water, and dried at 60 °C. Finally, the product was calcined in a N_2_ atmosphere at 400 °C for 3 h.

### 3.3. Synthesis of Zn_2_GeO_4_@xCeO_2_

First, 50 mg of uncalcined Zn_2_GeO_4_ crystals was dispersed by ultrasound in a mixed solution of 40 mL of water and 40 mL of ethanol. Next, 0.05 mmol Ce(NO_3_)_3_·6H_2_O and 0.1 mmol HMT were added sequentially. Then, the mixture was heated at 60 °C for 2 h and cooled to room temperature. The product was purified by centrifugation, washed three times with water, and dried at 60 °C. Finally, the product was calcined in a N_2_ atmosphere at 400 °C for 3 h. The prepared samples are named Zn_2_GeO_4_@0.05CeO_2_ based on the amount of added Ce(NO_3_)_3_·6H_2_O. Using the same method, Zn_2_GeO_4_@0.07CeO_2_ and Zn_2_GeO_4_@0.1CeO_2_ were prepared by controlling the amount of added Ce(NO_3_)_3_·6H_2_O and HMT, where x represents the amount of Ce added per 50 mg of Zn_2_GeO_4_. The corresponding amount of HMT is added proportionally to the amount of Ce(NO_3_)_3_·6H_2_O, maintaining a molar ratio of 2:1 between HMT and Ce(NO_3_)_3_·6H_2_O.

### 3.4. Characterization

The XRD patterns were obtained on an XRD-6000 (Shimadzu Corporation, Kyoto, Japan) X-ray diffractometer. The morphologies of the samples were measured by a field-emission scanning electron microscope on the Quanta 250FEG scanning electron microscope (FEI, Hillsboro, OR, USA). The XPS measurements were performed by a K-Alpha electron spectrometer (Thermo Fisher Scientific, Waltham, MA, USA). The quantification of liquid products was performed using NMR spectroscopy (Bruker Corporation, Billerica, MA, USA) with dimethyl sulfoxide as the internal standard. The UV-vis analysis was carried out on a UV-300 UV Visible Spectrophotometer (Shimadzu Corporation, Kyoto, Japan). The CO_2_-TPD experiments were conducted in a quartz tube reactor under a flow of 20 vol % of CO_2_/Ar (20 mL/min) over 100 mg of catalysts, and the desorption was conducted under a flow of N_2_ (20 mL/min). The photoluminescence (PL) emission spectra of samples were collected using an FLS-1000 luminescence spectrometer (Edinburgh Instruments, Edinburgh, UK). The in situ FT-IR spectra were measured through Fourier transform infrared spectroscopy (Bruker Corporation, MA, USA).

### 3.5. Photocatalytic Performance Measurements

A total of 25 mg of the photocatalyst samples and 20 mL of deionized water with 10 mL of trolamine were added into a 230 mL reactor (CEL-APR100H, China Education Au-light Technology Co. Ltd, Beijing, China) under ultrasonic dispersion. After that, high-purity CO_2_ gas was injected for 30 min to achieve a partial pressure of 1.0 atm. The reactor was irradiated by a 300 W Xe lamp (CEL-PUV300-T8, China Education Au-light Technology Co., Ltd., Beijing, China) with circulating cooling water to keep it at room temperature. After the irradiation reaction had occurred for 3 h, 1 mL of the evolved gas was pumped from the reactor and examined by a gas chromatograph.

For the cycle tests, the photocatalyst after reaction is centrifugally separated, washed with deionized water and ethanol sequentially, and then vacuum-dried prior to subsequent testing under the same conditions.

The gas product (CO, CH_4_, H_2_, C_2_) yield (μmol g^−1^ h^−1^) and product selectivity of gas product i were calculated using the following equations:

(1) $${Yield}_{i}=\frac{c_{i}\times (\frac{pV}{RT})}{Wt}\times 10^{6}$$

(2) $${Selectivity}_{i}=\frac{\;c_{i}\times (\frac{pV}{RT})\times number\;of\;electrons}{n_{CO}\times 2+n_{{CH}_{4}}\times 8+n_{H_{2}}\times 2+n_{C_{2}H_{6}}\times 14+n_{C_{2}H_{4}}\times 12}$$

Here, $c_{i}\;$(ppm) represents the molar concentration of i obtained by gas chromatography (GC), p (Pa) is the pressure inside the reaction system, V (m^3^) is the volume of the reactor, R is the universal gas constant, T (K) is the temperature under light irradiation, W (g) is the mass of the catalyst, t (h) is the reaction time, and $n_{i}$ is the mole number of i.

### 3.6. Photoelectrochemical Measurements

For the photoelectrochemical testing, 5 mg of the sample was added to a solution of 1000 μL of isopropanol and 50 μL of naphthol, sonicated for 40 min, and stirred for 5 min. After uniform dispersion, 80 μL of the dispersion solution was applied to an ITO glass slide to form a square area of 1 cm × 1 cm and dried for 15 min. Photoelectrochemical testing was conducted using a CH650E electrochemical workstation in a three-electrode system, with the electrolyte being 0.5 M sodium sulfate solution. A Ag/AgCl electrode and Pt electrode were used as the reference electrode and counter electrode, respectively. The ITO glass sheet coated with the sample was used as the working electrode. The electrochemical impedance test was conducted under a forward bias voltage of the open circuit voltage, amplitude of 0.005 V, and frequency range of 0.001–100,000 Hz. The photocurrent test was conducted under a bias voltage of 0 V, using a 300 W xenon lamp (CEL-PUV300-T8, China Education Au-light Technology Co. Ltd., Beijing, China) to illuminate the working electrode, and turning on/off the light source every 30 s for testing. Mott–Schottky measurements were also tested at frequencies of 1000/2000/3000 Hz.

## 4. Conclusions

In this work, Zn_2_GeO_4_@CeO_2_ nanorods with a special core@shell structure have been successfully fabricated through a simple self-assembly method. By precisely controlling the amount of Ce(NO_3_)_3_·6H_2_O, a series of rod-shaped Zn_2_GeO_4_@CeO_2_ composites with varying amounts of CeO_2_ can be obtained. The photocatalytic CO_2_ reduction experiments demonstrated that an appropriate CeO_2_ thickness is beneficial for catalytic performance, in which Zn_2_GeO_4_@0.07CeO_2_ exhibited the highest activity with a CO yield of 1190.9 μmol g^−1^ h^−1^. Further characterization indicates that Zn_2_GeO_4_ and CeO_2_ formed a Type-I heterojunction. The excited electrons will transfer from Zn_2_GeO_4_ to CeO_2_, which could extend the carrier lifetime. Furthermore, the CO_2_ TPD results demonstrated the enhanced CO_2_ adsorption capacity of the CeO_2_ shell. Finally, the in situ FT-IR spectra confirmed the high CO selectivity of Zn_2_GeO_4_@0.07CeO_2_ and demonstrated that the CeO_2_ coating modifies the pathway selectivity of CO_2_ adsorption and reduction, suppresses the formation of byproduct intermediates, and thereby achieves enhanced CO selectivity.

## Data Availability

The data that support the findings of this study are available from the corresponding author upon reasonable request.

## Supplementary Materials

The following supporting information can be downloaded at: https://www.mdpi.com/article/10.3390/molecules30102205/s1, Figure S1: SEM images of the as-obtained Zn_2_GeO_4_@CeO_2_ samples: (a,b) Zn_2_GeO_4_, (c,d) CeO_2_ nanoparticles, (e,f) Zn_2_GeO_4_@0.05CeO_2_, (g,h) Zn_2_GeO_4_@0.07CeO_2_, and (i,j) Zn_2_GeO_4_@0.1CeO_2_; Figure S2: SEM images of the as-obtained Zn_2_GeO_4_@CeO_2_ samples: (a) Zn_2_GeO_4_@0.05CeO_2_, (b) Zn_2_GeO_4_@0.07CeO_2_, and (c) Zn_2_GeO_4_@0.1CeO_2_; Figure S3: HRTEM images of the as-obtained Zn_2_GeO_4_@0.07CeO_2_; Figure S4: XRD patterns of Zn_2_GeO_4_@0.05CeO_2_, Zn_2_GeO_4_@0.07CeO_2_, and Zn_2_GeO_4_@0.1CeO_2_; Figure S5: EDS mapping images of Zn_2_GeO_4_@xCeO_2_: (a) Zn_2_GeO_4_@0.05CeO_2_, (b) Zn_2_GeO_4_@0.07CeO_2_, and (c) Zn_2_GeO_4_@0.1CeO_2_; Figure S6: EDS line scan images of Zn_2_GeO_4_@xCeO_2_: (a,d) Zn_2_GeO_4_@0.05CeO_2_, (b,e) Zn_2_GeO_4_@0.07CeO_2_, and (c,f) Zn_2_GeO_4_@0.1CeO_2_; Figure S7: EPR patterns of CeO_2_; Figure S8: Simulated ^1^H-NMR spectra for the liquid-phase products of the Zn_2_GeO_4_@0.07CeO_2_ system after reaction; Figure S9: CO yield of Zn_2_GeO_4_@0.07CeO_2_ under different conditions; Figure S10: SEM images of Zn_2_GeO_4_@0.07CeO_2_ (a) before and (b) after reaction; Figure S11: XRD patterns of Zn_2_GeO_4_@0.07CeO_2_ before and after the reaction; Figure S12: XPS spectra of Zn_2_GeO_4_@0.07CeO_2_ before and after the reaction. (a) Zn 2p; (b) Ge 3d; (c) O 1s; (d) Ce 3d; Figure S13: (a) UV-vis spectra, (b) Tauc spectra, and (c) Mott–Schottky curves of Zn_2_GeO_4_@xCeO_2_; Figure S14: Band structures of Zn_2_GeO_4_@0.07CeO_2_ (pH = 7, vs. NHE); Figure S15: PL spectra of Z_2_GeO_4_, CeO_2_, and Zn_2_GeO_4_@xCeO_2_; Table S1: The relative concentrations of Ce^3+^ and Ce^4+^ calculated by XPS spectra; Table S2: Yield of products in photocatalytic CO_2_ reduction; Table S3: Comparison with similar catalysts; Table S4: Comparison with similar catalysts without using hole scavengers.
