# Supplementary material for: Zn2GeO4@CeO2 Core@Shell Nanorods for Efficient Photocatalytic CO2 Reduction
Source: Molecules. 2025 May 18;30(10):2205. doi: 10.3390/molecules30102205 (PMC12114070; doi:10.3390/molecules30102205)
Supplement: Supplementary file 1 [file molecules-30-02205-s001.zip › molecules-3635486-supplementary.pdf]

Supporting Information

Zn_2GeO_4@CeO_2 Core@Shell Nanorods for Efficient Photocatalytic CO_2 Reduction

Jin Sun [1,2,†], Yunxia Bai [1,2,†], Xilan Feng [3,4,*], Dapeng Liu [1,2,*] and Yu Zhang [1,2]

[1] Hangzhou International Innovation Institute, Beihang University, Hangzhou 311115, China;
20271015@buaa.edu.cn (J.S.); baiyunxia@buaa.edu.cn (Y.B.); jade@buaa.edu.cn (Y.Z.)
[2] School of Chemistry, Beihang University, Beijing 100191, China
[3] School of Chemistry and Chemical Engineering, Shaoxing University, Shaoxing 312000, China
[4] Zhejiang Key Laboratory of Functional Ionic Membrane Materials and Technology for Hydrogen Production, Shaoxing 312000, China
* Correspondence: 2025000022@usx.edu.cn (X.F.); liudp@buaa.edu.cn (D.L.)
† These authors contributed equally to this work.

Supporting Figures

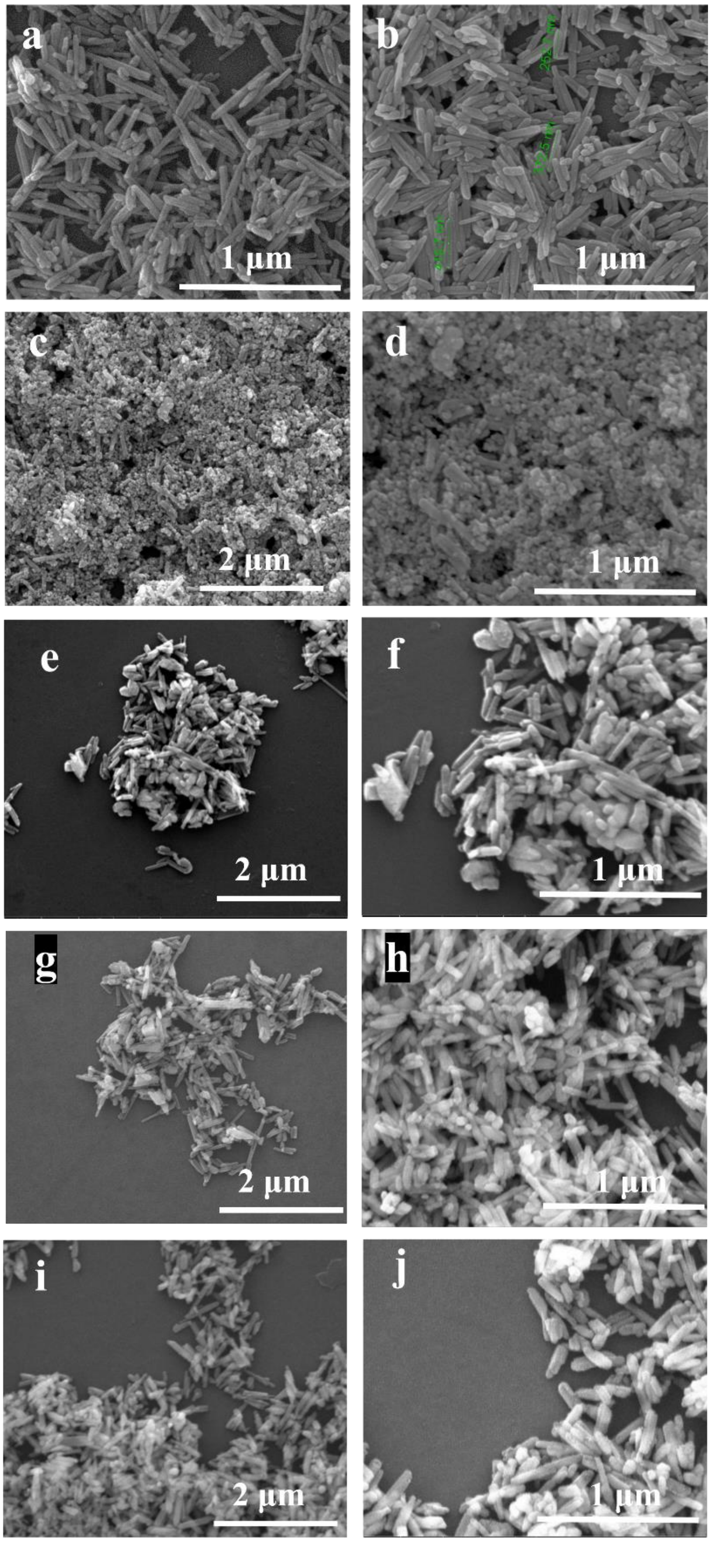

Figure S1. SEM images of the as-obtained Zn_2GeO_4@CeO_2 samples: (**a** and **b**) Zn_2GeO_4, (**c** and **d**) CeO_2 nanoparticles, (**e** and **f**) Zn_2GeO_4@0.05CeO_2, (**g** and **h**) Zn_2GeO_4@0.07CeO_2, and (**i** and **j**) Zn_2GeO_4@0.1CeO_2.

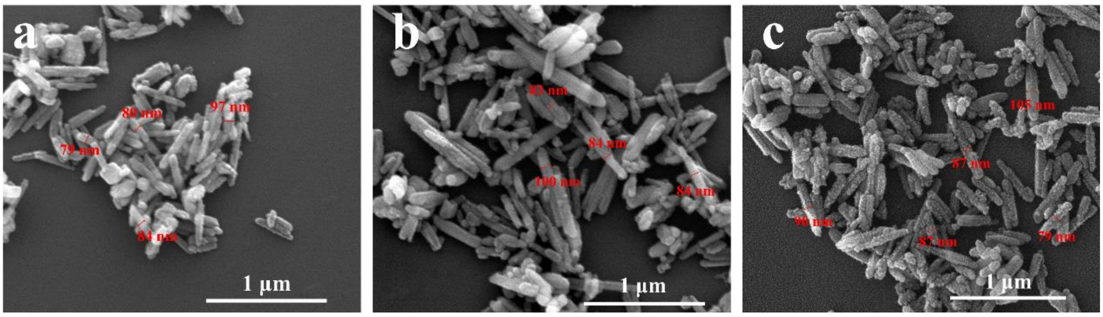

Figure S2. SEM images of the as-obtained $Zn_2GeO_4@CeO_2$ samples: (**a**) $Zn_2GeO_4@0.05CeO_2$, (**b**) $Zn_2GeO_4@0.07CeO_2$, and (**c**) $Zn_2GeO_4@0.1CeO_2$.

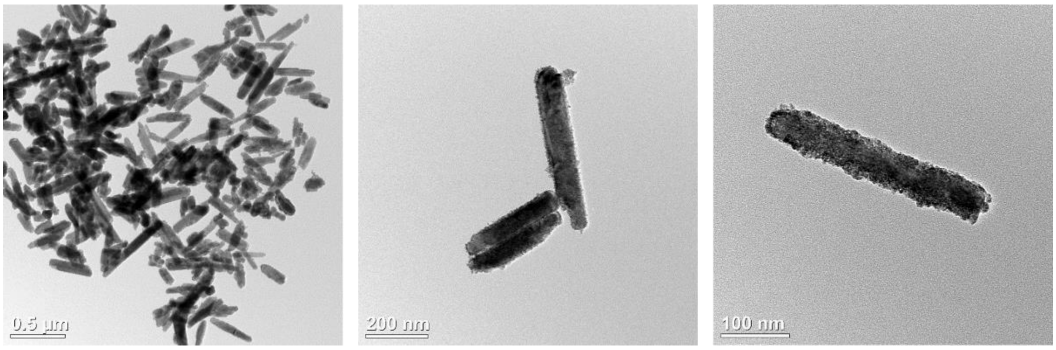

Figure S3. HRTEM images of the as-obtained $Zn_2GeO_4@0.07CeO_2$.

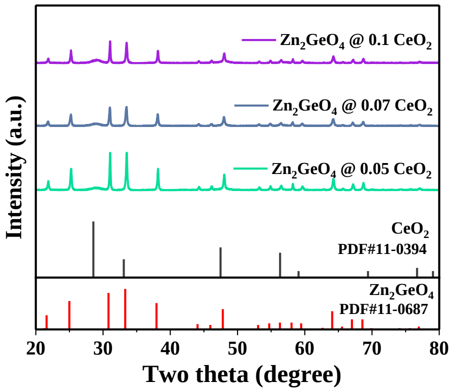

Figure S4. XRD patterns of $Zn_2GeO_4@0.05CeO_2$, $Zn_2GeO_4@0.07CeO_2$, and $Zn_2GeO_4@0.1CeO_2$.

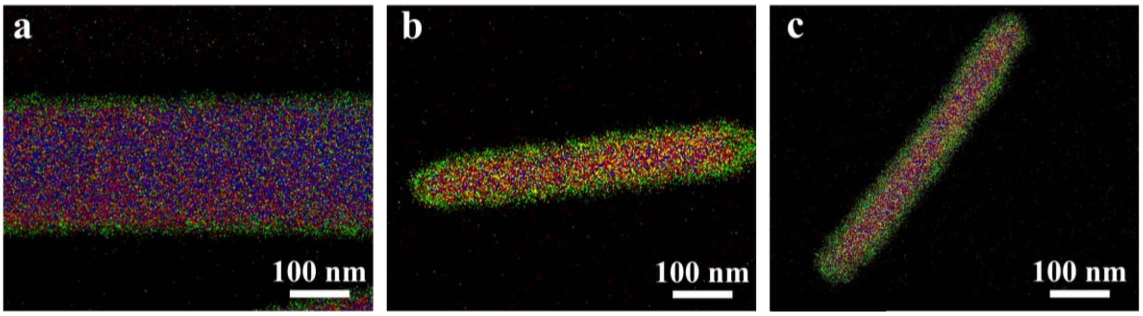

Figure S5. EDS mapping images of $Zn_2GeO_4@xCeO_2$: (**a**) $Zn_2GeO_4@0.05CeO_2$, (**b**) $Zn_2GeO_4@0.07CeO_2$, and (**c**) $Zn_2GeO_4@0.1CeO_2$.

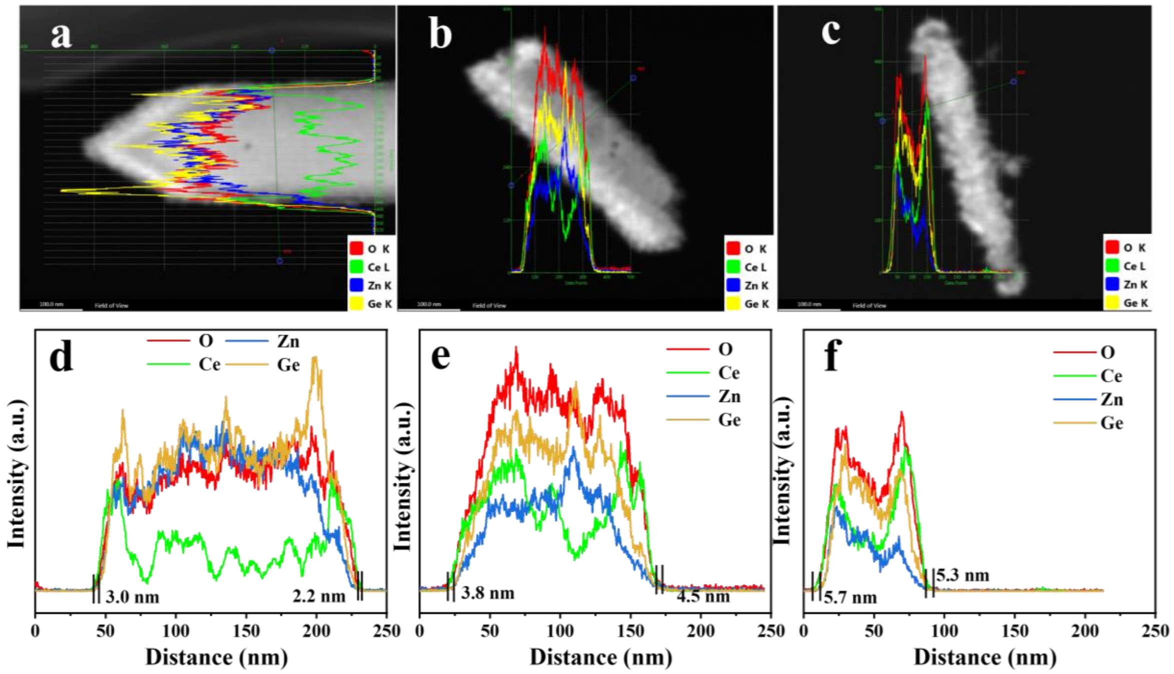

Figure S6. EDS line scan images of $Zn_2GeO_4@xCeO_2$: (**a** and **d**) $Zn_2GeO_4@0.05CeO_2$, (**b** and **e**) $Zn_2GeO_4@0.07CeO_2$, and (**c** and **f**) $Zn_2GeO_4@0.1CeO_2$.

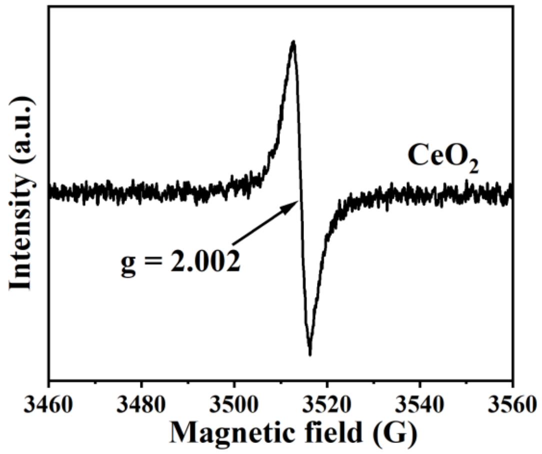

Figure S7. EPR patterns of CeO_2.

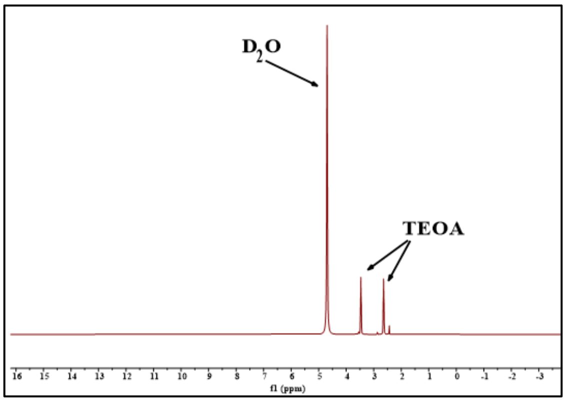

Figure S8. Simulated ^{1}H-NMR spectra for the liquid-phase products of the Zn_2GeO_4@0.07CeO_2 system after reaction.

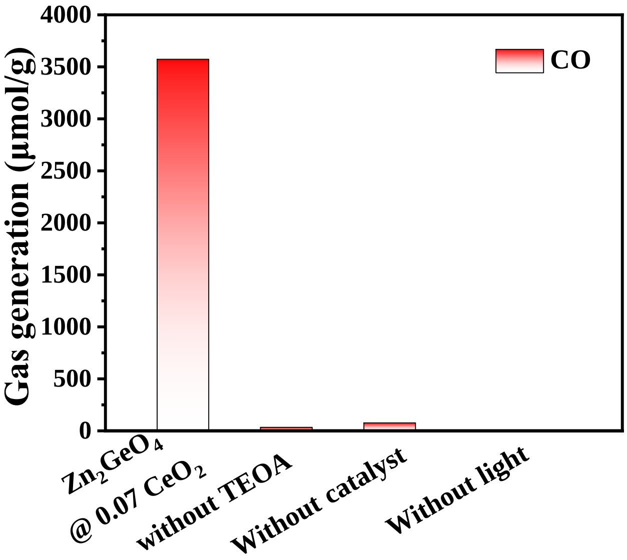

Figure S9. CO yield of Zn_2GeO_4@0.07CeO_2 under different conditions.

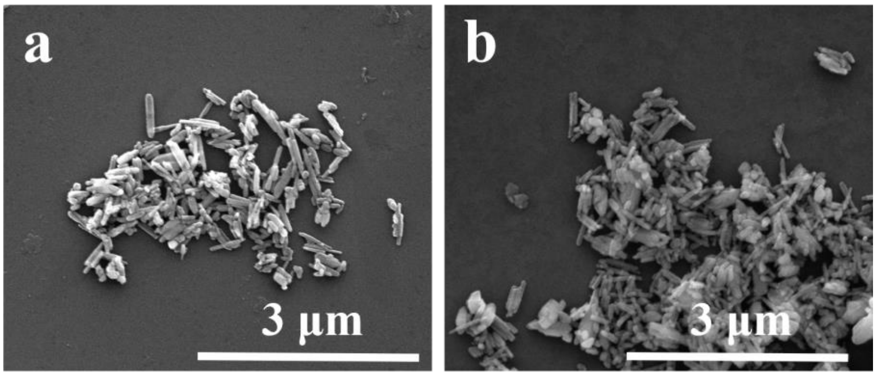

Figure S10. SEM images of Zn_2GeO_4@0.07CeO_2 (**a**) before and (**b**) after reaction.

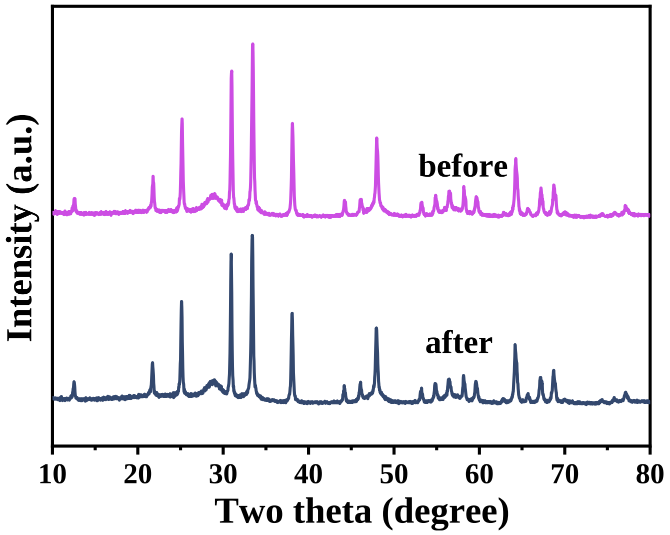

Figure S11. XRD patterns of $Zn_2GeO_4@0.07CeO_2$ before and after the reaction.

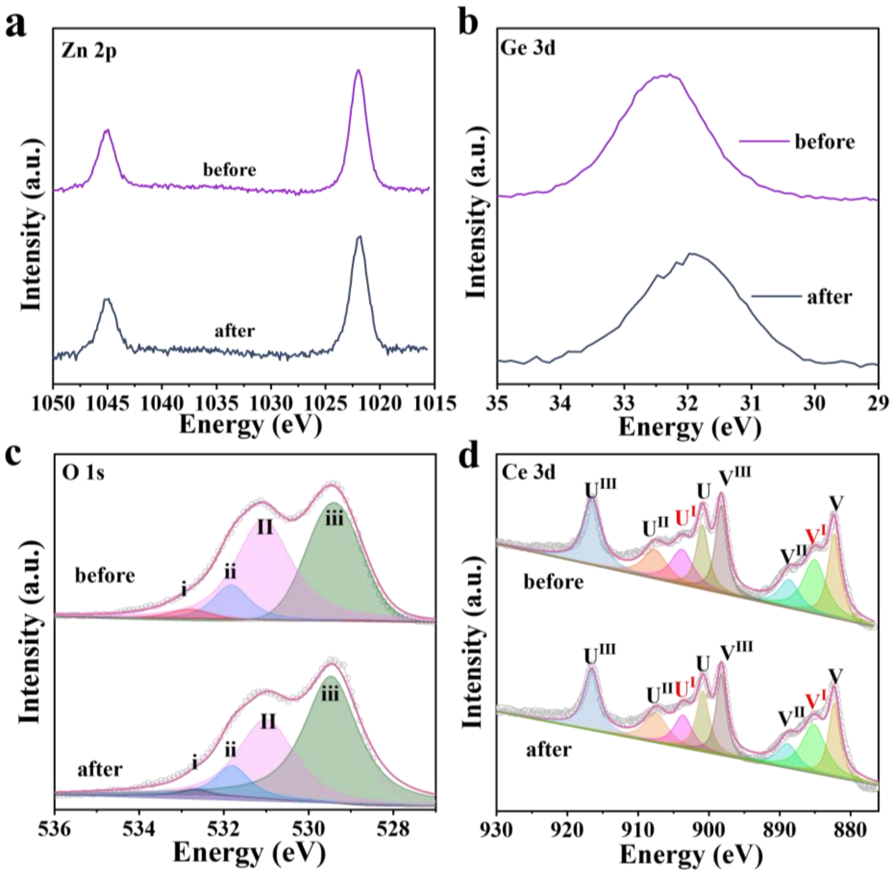

Figure S12. XPS spectra of $Zn_2GeO_4@0.07CeO_2$ before and after the reaction. (**a**) Zn 2p, (**b**) Ge 3d; (**c**) O 1s; (**d**) Ce 3d.

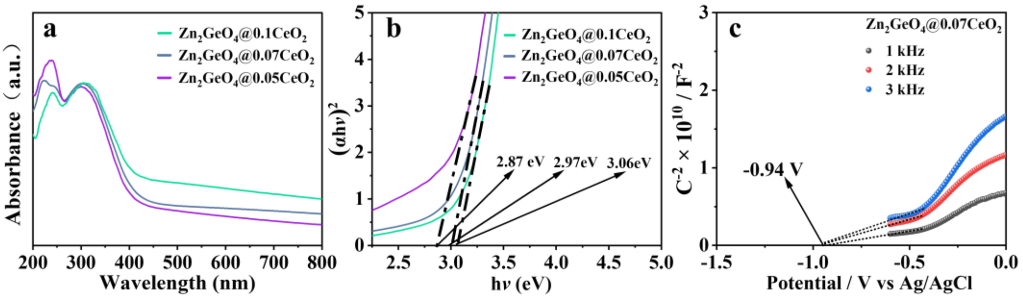

Figure S13. (**a**) UV-vis spectra (**b**) Tauc spectra and (**c**) Mott-Schottky curves of Zn_2GeO_4@xCeO_2.

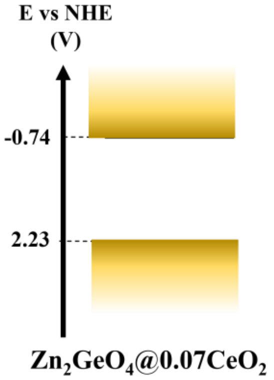

Figure S14. Band structures of Zn_2GeO_4@0.07CeO_2 (pH = 7, vs NHE).

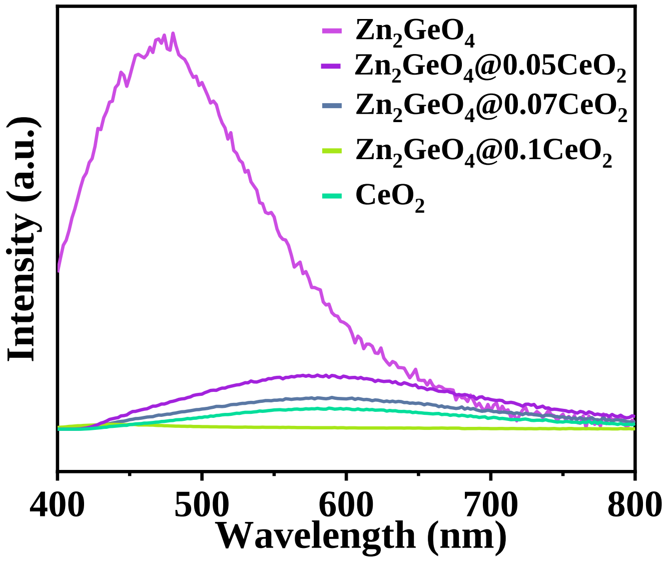

Figure S15. PL spectra of Zn_2GeO_4, CeO_2 and Zn_2GeO_4@xCeO_2.

Supporting Tables

Table S1. The relative concentrations of Ce^{3+} and Ce^{4+} calculated by XPS spectra.

Samples	Ce^{3+}/ (Ce^{3+} + Ce^{4+}) /%	Ce^{3+}/ (Ce^{3+} + Ce^{4+}) /%
**Zn_2GeO_4@0.07CeO_2**	35.70	64.30
**CeO_2**	25.84	74.16

The peaks at 884.97 eV (V^{I}) and 903.71 eV (U^{I}) could be located to Ce^{3+}. The other six fitting peaks at the binding energies of 882.34 eV (V), 888.58 eV (V^{II}), 898.23 eV (V^{III}), 900.91 eV (U), 907.55 eV (U^{II}), and 916.63 eV (U^{III}) can be indexed to Ce^{4+}.The above results could indicate the coexistence of Ce^{3+} and Ce^{4+} species in Zn_2GeO_4@0.07CeO_2 and pure CeO_2 samples. To estimate Ce^{3+} content, the relative area of the V^{I} and U^{I} were calculated by the following equation:

$$\mathrm{Ce(III)\%}= \frac{100 \times S_{(U^{'})+(V^{'})}}{\sum S_{(U)+(V)}}\%$$

where $\sum S_{(U)+(V)}$ represents the total area of V, V^{I}, V^{II}, V^{III}, U, U^{I}, U^{II}, and U^{III} in the Ce 3d region, $S_{(U^{'})+(V^{'})}$ represents the total area of U^{I} and V^{I}.

Table S2. Yield of products in photocatalytic CO_2 reduction.

**Catalyst**	**Yield (μmol g^{-1})**				
	**CO**	**CH_4**	**C_2H_4**	**C_2H_6**	**H_2**
**Zn_2GeO_4**	1440.1	1023.6	1147.1	27.1	1161.5
**Zn_2GeO_4@0.05CeO_2**	1860.7	639.1	843.0	7.0	1199.8
**Zn_2GeO_4@0.07CeO_2**	3572.8	334.8	63.7	31.1	1245.6
**Zn_2GeO_4@0.1CeO_2**	1779.6	338.3	120.0	19.9	689.3
**CeO_2**	117.8	15.3	0.2	0	157.8

Table S3. Comparison with similar catalysts.

Material	Catalytic conditions	Reduction products (μmol g^{-1} h^{-1})	Reference
**Zn_2GeO_4@ 0.07CeO_2**	300 W Xe lamp TEOA	CO 1190.94	This work
**Zn_2GeO_4**	300 W Xe lamp TEOA	CO 380.05	This work
**CoS@CdZnS -DETA**	300 W Xe lamp ($\lambda \geq 420$ nm) bpy/MeCN/ TEOA/ $CoCl_2 \cdot 6H_2O$	CO 4706.6	[1]
**(TS)-$Zn_xCd_{1-x}S$–Co_1**	300 W Xe lamp bpy/MeCN/ TEOA/ $CoCl_2 \cdot 6H_2O$	CO 7629.7	[2]
**Ni/ SOM-ZIF-8**	300 W Xe lamp ($\lambda \geq 400$ nm) TEOA/MeCN/ $[Ru(bpy)_3]Cl_2$	CO 4200	[3]
**$CdIn_2S_4$/ $ZnIn_2S_4$-3**	300 W Xe lamp ($\lambda \geq 400$ nm) $KHCO_3$/bpy/MeCN/ TEOA/ $CoCl_2 \cdot 6H_2O$	CO 1194.5	[4]
**Au/TZO**	300 W Xe lamp TEOA/MeCN	CO 260.6	[5]
**CuO_x/p-ZnO**	300 W Xe lamp TEA	CO 27.3	[6]
**$Ce_{1-x}Ti_xO_2$/ CeO_2-0.8**	300 W Xe lamp TEOA/MeCN/ $[Ru(bpy)_3]Cl_2$	CO 12.4 CH_4 23.6	[7]

Table S4. Comparison with similar catalysts without using hole scavengers.

**Zn_2GeO_4@ 0.07CeO_2**	**300 W Xe lamp**	**CO 10.90**	**This work**
Zn_2GeO_4	300 W Xe lamp	CO 5.20	[8]
**Mn 1% - Zn_2GeO_4**	300 W Xe lamp	CO 22.87	[8]
**Atomically thin Zn_2GeO_4**	300 W Xe lamp	CO 20.81	[9]
**Vo-rich Zn_2GeO_4 nanobelts**	100 W/cm^2 Xe lamp	CO 25.13	[10]
**Zn_2GeO_4/ Mg-MOF-74**	300 W Xe lamp	CO 1.45	[11]
**ZIS@NOH/N O**	300 W Xe lamp ($\lambda \geq 420$ nm)	CO 133.74	[12]
**BSB@SIS-3**	300 W Xe lamp	CO 35.63	[13]
**CuO@In_2O_3**	300 W Xe lamp ($\lambda \geq 420$)	CO 500.46	[14]

References

1. Su, B.; Zheng, M.; Lin, W.; Lu, X. F.; Luan, D.; Wang, S.; Lou, X. W. (David). S-Scheme Co_9S_8@$Cd_{0.8}Zn_{0.2}S$-DETA Hierarchical Nanocages Bearing Organic CO_2 Activators for Photocatalytic Syngas Production. *Adv. Energy Mater.* **2023**, *13*, 2203290.
2. Zeng, R.; Liu, T.; Qiu, M.; Tan, H.; Gu, Y.; Ye, N.; Dong, Z.; Li, L.; Lin, F.; Sun, Q.; Zhang, Q.; Gu, L.; Luo, M.; Tang, D.; Guo, S. High-Volumetric Density Atomic Cobalt on Multishell $Zn_xCd_{1-x}S$ Boosts Photocatalytic CO_2 Reduction. *J. Am. Chem. Soc.* **2024,** *146*, 9721–9727.
3. Liu, Z.; Chen, Z.; Li, M.; Li, J.; Zhuang, W.; Yang, X.; Wu, S.; Zhang, J. Construction of Single Ni Atom-Immobilized ZIF-8 with Ordered Hierarchical Pore Structures for Selective CO_2 Photoreduction. *ACS Catal.* **2023**, *13*, 6630-6640.
4. Zhang, G.; Wang, Z.; He, T.; Wu, J.; Zhang, J.; Wu, J. Rationally Design and In-Situ Fabrication of Ultrasmall Pomegranate-like $CdIn_2S_4$/$ZnIn_2S_4$ Z-Scheme Heterojunction with Abundant Vacancies for Improving CO_2 Reduction and Water Splitting. *Chem. Eng. J.* **2022**, *442*, 136309.
5. Huang, N.; Li, B.; Wu, D.; Chen, Z.; Shao, B.; Chen, D.; Zheng, Y.; Wang, W.; Yang, C.; Gu, M.; Li, L.; Xu, Q. Crystal Engineering of MOF-Derived Bimetallic Oxide Solid Solution Anchored with Au Nanoparticles for Photocatalytic CO_2 Reduction to Syngas and C_2 Hydrocarbons. *Angew. Chem. Int. Ed* **2024**, *63*, e202319177.
6. Wang, W.; Deng, C.; Xie, S.; Li, Y.; Zhang, W.; Sheng, H.; Chen, C.; Zhao, J. Photocatalytic C–C

Coupling from Carbon Dioxide Reduction on Copper Oxide with Mixed-Valence Copper(I)/Copper(II). *J. Am. Chem. Soc.* **2021**, *143*, 2984-2993.

7. Wei, J.; Mu, X.; Hu, Y.; Liu, L.; Wu, X.; Liu, Q.; Zhang, T.; Peng, Y.; Cao, J.; Yan, C.; Tang, Y. A General Preparation of Solid Solution-Oxide Heterojunction Photocatalysts through Metal–Organic Framework Transformation Induced Pre-nucleation. *Angew. Chem. Int. Ed.* **2023**, *62*, e202302986.
8. Ma, Z.; Liu, X.; Wang, X.; Luo, Z.; Li, W.; Nie, Y.; Pei, L.; Mao, Q.; Wen, X.; Zhong, J. Manipulating the D-Band Center Enhances Photoreduction of CO_2 to CO in Zn_2GeO_4 Nanorods. *Chem. Eng. J.* **2023**, *468*, 143569.
9. Yuan, Y.; Dai, H.; Chi, H.; Gao, W.; Liu, Q.; Ding, C.; Shen, Y.; Tang, Z.; Zhuang, C.; Yang, Y.; Zhang, Y.; Zou, Z.; Zhou, Y. Atomically Thin Zn_2GeO_4 Nanoribbons: Facile Synthesis and Selective Photocatalytic CO_2 Reduction toward CO. *ACS Mater. Lett.* **2022**, *4*, 2631–2637.
10. Zhu, J.; Shao, W.; Li, X.; Jiao, X.; Zhu, J.; Sun, Y.; Xie, Y. Asymmetric Triple-Atom Sites Confined in Ternary Oxide Enabling Selective CO_2 Photothermal Reduction to Acetate. *J. Am. Chem. Soc.* **2021**, *143*, 18233–18241.
11. Zhao, H.; Wang, X.; Feng, J.; Chen, Y.; Yang, X.; Gao, S.; Cao, R. Synthesis and Characterization of Zn_2GeO_4/Mg-MOF-74 Composites with Enhanced Photocatalytic Activity for CO_2 Reduction. *Catal. Sci. Technol.* **2018**, *8*, 1288.
12. Wang, J.; Huang, L.; Sun, B.; Zhang, H.; Hou, D.; Qiao, X.; Ma, H.; Li, D.-S. Efficient Photothermal Catalytic CO_2 Reduction over in Situ Construction $ZnIn_2S_4$@$Ni(OH)_2$/NiO Z-Scheme Heterojunction. *Chem. Eng. J.* **2024**, *479*, 147719.
13. Jia, W.; Xiong, R.; Sun, Y.; Xiao, Y.; Cheng, B.; Lei, S. A Well-Designed Hierarchical $Bi_{19}S_{27}Br_3$ nanorods @$SnIn_4S_8$ Nanosheet Core–Shell S-Scheme Heterostructure for Robust Photothermal-Assisted Photocatalytic CO_2 Reduction. *J. Mater. Chem. A* **2024**, *12*, 4513–4524.
14. Liu, X.; Wu, Y.; Li, Y.; Yang, X.; Ma, Q.; Luo, J. MOF-on-MOF-Derived CuO@In_2O_3 S-Scheme Heterojunction with Core–Shell Structure for Efficient Photocatalytic CO_2 Reduction. *Chem. Eng. J.* **2024**, *485*, 149855.
